# Dexmedetomidine Protects Rat Liver against Ischemia-Reperfusion Injury Partly by the α_2A_-Adrenoceptor Subtype and the Mechanism Is Associated with the TLR4/NF-κB Pathway

**DOI:** 10.3390/ijms17070995

**Published:** 2016-06-23

**Authors:** Yiheng Wang, Shan Wu, Xiaofang Yu, Shaoli Zhou, Mian Ge, Xinjin Chi, Jun Cai

**Affiliations:** 1 Department of Anesthesiology, The Third Affiliated Hospital, Sun Yat-sen University, 600 Tianhe Road, Guangzhou 510630, China; wyheng1202@126.com (Y.W.); wsbiosci@sina.com (S.W.); doctorzsl@sina.com (S.Z.); gmsunyatsen@sina.com (M.G.); 2 Department of Anesthesiology, The First Affiliated Hospital, University of South China, Hengyang 421001, China; 3 Department of Anesthesiology, Fujian Provincial Hospital, Fuzhou 350000, China; yxfanesthesia@hotmail.com

**Keywords:** dexmedetomidine, ischemia-reperfusion injury, liver transplantation, TLR4/NF-κB, α_2A_-adrenoceptor subtype

## Abstract

Toll-like receptor 4 (TLR4)/nuclear factor kappa B (NF-κB) signaling plays a dominant role in the pathogenesis of liver ischemia-reperfusion (IR) injury. Dexmedetomidine (Dex) protects the liver against IR injury via α_2_-adrenoceptor activation, but the contribution of TLR4 signaling remains unknown. The authors aimed to examine whether pretreatment with Dex produces hepatic protection and investigate the influence of Dex on TLR4/NF-κB signaling. Dex was given via intraperitoneal injection 30 min prior to orthotopic autologous liver transplantation (OALT) in rats, and three α_2_-adrenoceptor antagonists including atipamezole (a nonselective α_2_ receptor blocker), ARC-239 (a specific α_2B/C_ blocker) and BRL-44408 (a specific α_2A_ blocker) were injected intraperitoneally 10 min before Dex administration. Histopathologic evaluation of the liver and the measurement of serum alanine aminotransferase activity, TLR4/NF-κB expression in the liver, and pro-inflammatory factors (serum tumor necrosis factor-α, interleukin-1β and hepatic myeloperoxidase) concentrations were performed 8 h after OALT. Dex ameliorated liver injury after OALT probably by suppressing the TLR4/NF-κB pathway and decreasing inflammatory mediator levels. The protective effects of Dex were reversed by atipamezole and BRL-44408, but not by ARC-239, suggesting that these effects were mediated in part by the α_2A_ subtype. In conclusion, Dex attenuates liver injury partly via the α_2A_-adrenoceptor subtype, and the mechanism is due to the suppression of the TLR4/NF-κB pathway.

## 1. Introduction

Ischemia-reperfusion (IR) injury in the liver remains a major problem during liver transplantation and resection surgery [1,2]. For liver transplant recipients, the consequences of IR injury are significant. In addition to early hepatic dysfunction or non-function, IR injury leads to a higher incidence of acute rejection, longer hospitalization, and lower long-term graft survival rates [3,4].

Liver IR involves a complex interaction of events that include Kupffer cell activation, neutrophil infiltration, the generation of reactive oxygen species, and the release of cytokines (tumor necrosis factor-α (TNF-α) and interleukin-1β (IL-1β), etc.), ultimately followed by endothelial cell and hepatocyte death [2,5,6]. The Toll-like receptor (TLR) family, particularly Toll-like receptor 4 (TLR4), is one of the molecular mechanisms mediating the deleterious effects that occur during liver IR injury [6,7,8]. TLRs are widely expressed on the tissue of the liver, such as Kupffer cells, hepatocytes, and hepatic stellate cells [9]. Triggering the TLR pathway leads to the activation of nuclear factor kappa B (NF-κB) and subsequently upregulates the expression of inflammatory genes and inflammatory factors (i.e., TNF-α, IL-1β, and IL-6 (interleukin-6)) [7,8,9,10]. Drugs inhibiting the activity of the TLR inflammatory system afford potential beneficial effects for the liver, as do transgenic methods of blocking TLR4/NF-κB-related genes [6,11,12].

Dexmedetomidine (Dex), a potent and highly selective α_2_-adrenoceptor (α_2_-AR) agonist, is widely used for sedation in intensive care units and as an anesthetic adjunct. We [13,14] and others [15,16] found that Dex attenuates IR injury of the lung and kidney, partly through inhibiting the TLR4/NF-κB inflammatory circuit [12,13,14,15]. Regarding the liver, Dex has been found to be a protective agent against hepatic lipid peroxidation and histological damage in sepsis and in IR animal models [17,18,19]; however, the contribution of TLR4 signaling remains unknown. Furthermore, Dex is a complete α_2_-AR agonist with remarkable binding capacity for all three subtypes (α_2A_, α_2B_, and α_2C_) of the human α_2_-AR [20]. Most of the effects of Dex, such as the analgesic, sedative, hypotensive, and potential neuroprotective effects, are mediated by the α_2A_-adrenoceptor subtype [21,22,23]. Therefore, we hypothesized that Dex protects against IR injury of the liver by activating the α_2A_-adrenoceptor subtype, and the mechanism is due to the suppression of the TLR4/NF-κB inflammatory circuit.

## 2. Results

### 2.1. Dexmedetomidine Reduces Pathological Liver Damage in Rats Undergoing Orthotopic Autologous Liver Transplantation (OALT)

**Figure 1:**
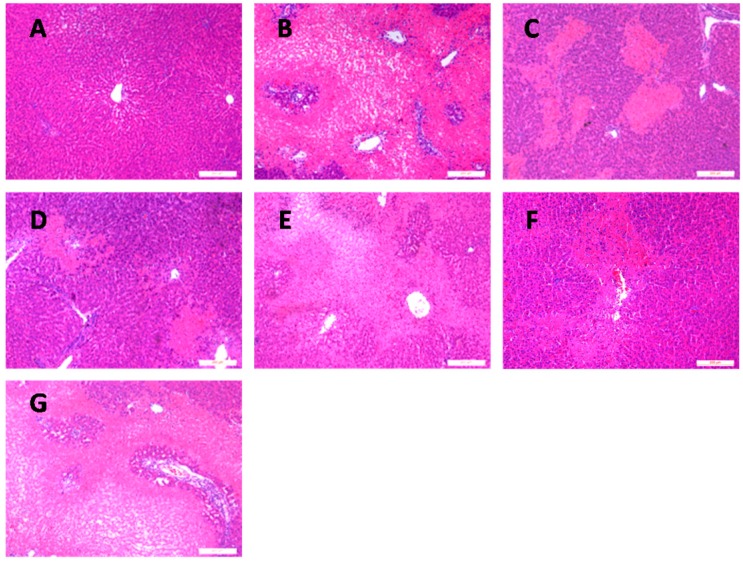
Histological changes in the liver of each group. Representative microphotographs taken from group S (**A**); group M (**B**); group D1 (**C**); group D2 (**D**); group B1 (**E**); group B2 (**F**); and group B3 (**G**). (H and E stain, 100× magnification; scale bars, 200 μm).

**Figure 2:**
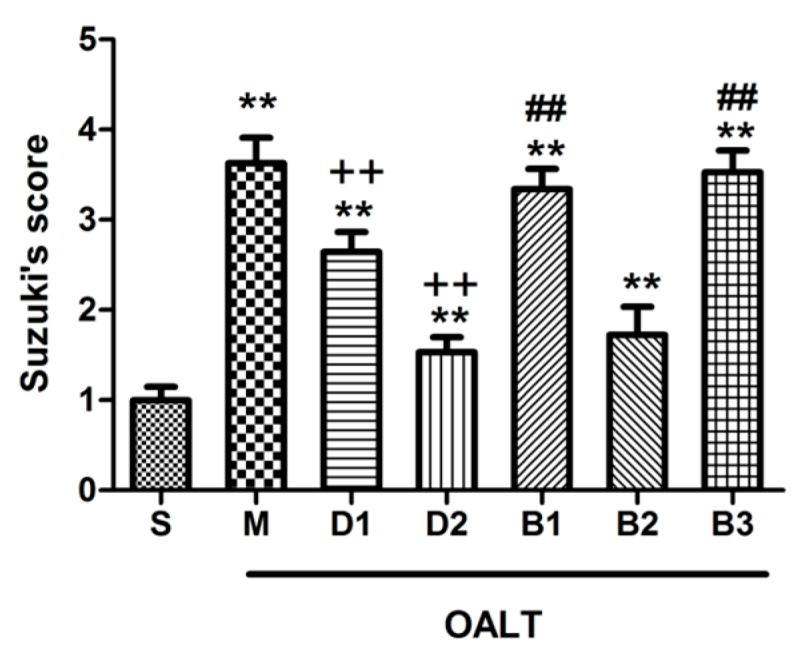
Quantification of histological scoring. The data are expressed as the means ± SD (*n* = 8). ** *p* < 0.01, vs. group S; ^++^ *p* < 0.01, vs. group M; ^##^ *p* < 0.01, vs. group D2.

In the current study, livers that underwent orthotopic autologous liver transplantation (OALT) displayed severe edema, lobular distortion, and sinusoidal congestion with necrosis area of over 70% (Figure 1B). In contrast, the Suzuki’s score and necrotic hepatocellular areas were significantly decreased with Dex preconditioning. In particular, Dex preconditioning yielded more beneficial hepatic alterations at 50 μg/kg than at 10 μg/kg (*p* < 0.01, group D1 or group D2 vs. group M; Figure 1C,D and Figure 2). Thus, Dex attenuated liver IR injury in a dose-dependent manner.

To determine which subtypes (α_2A_, α_2B_, or α_2C_) primarily contributed to the hepatoprotection by Dex, one of three α_2_-AR-related antagonists was pre-injected before administering the Dex. As a non-selective α_2_ subtype antagonist, atipamezole was found to block the protective effect of Dex on liver damage (Figure 1E and Figure 2). BRL-44408, the α_2A_ subtype-preferring antagonist, completely reversed the beneficial hepatic effect of Dex (Figure 1G and Figure 2); however, ARC-239 (the α_2B/C_ subtype-preferring antagonist) could not (Figure 1F and Figure 2).

### 2.2. Dexmedetomidine Improves Liver Function in Rats Undergoing OALT

**Figure 3:**
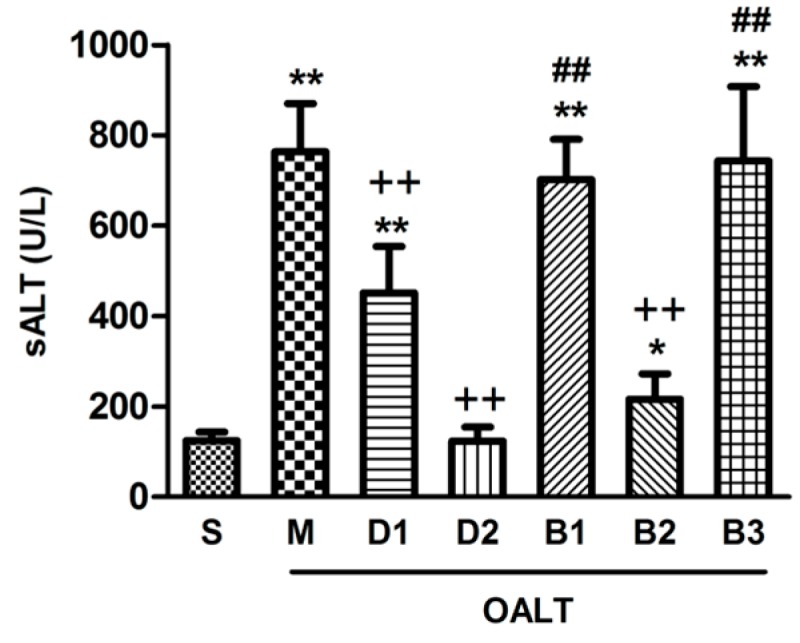
Serum alanine aminotransferase (sALT, U/L) level. The data are expressed as the means ± SD (*n* = 6–7). * *p* < 0.05, ** *p* < 0.01, vs. group S; ^++^ *p* < 0.01, vs. group M; ^##^ *p* < 0.01, vs. group D2.

Twenty minutes of ischemia followed by eight hours of reperfusion during OALT significantly increased serum alanine aminotransferase (sALT) levels (*p* < 0.01, group M vs. group S). In contrast, with Dex pre-injection, the sALT level decreased by 80% and 40% in groups D2 and D1, respectively (*p* < 0.01, group D1 and group D2 vs. group M). The pre-injection of atipamezole and BRL-44408 completely blocked the improvement of liver function with Dex (*p* < 0.01, group B1 and group B3 vs. group D2), whereas ARC-239 had no effect (*p* > 0.05, group B2 vs. group D2; Figure 3).

### 2.3. Dexmedetomidine Down-Regulates the Expression of the TLR4 and NF-κB Proteins in Liver Tissue

**Figure 4:**
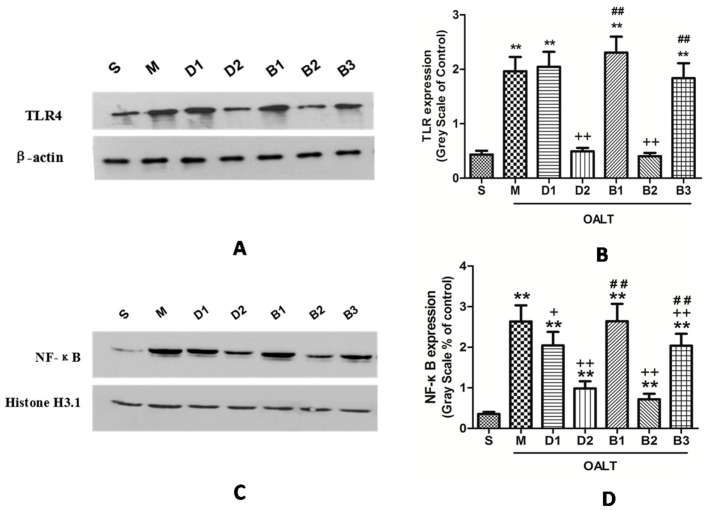
Expression of the Toll-Like Receptor 4 (TLR4) and NF-κB proteins in liver tissue of the different groups. (**A**) representative Western blotting for TLR4 in total proteins; (**B**) quantification analysis of TLR4 protein expressions by reference to β-actin content; (**C**) representative Western blotting for NF-κB p65 in nuclear proteins; and (**D**) quantification analysis of NF-κB p65 protein expressions by reference to Histone H3.1 content. The data are expressed as the means ± SD (*n* = 5–7). ** *p* < 0.01, vs. group S; ^+^ *p* < 0.05, ^++^ *p* < 0.01, vs. group M; ^##^ *p* < 0.01, vs. group D2.

As depicted in Figure 4, Hepatic TLR4 protein expression was significantly elevated after OALT. Pretreatment with Dex at 50 µg/kg significantly increased the expression of the TLR4 protein (*p* < 0.01, group M vs. group S), while Dex at 10 µg/kg did not (*p* > 0.05, group D1 vs. group M). This effect was blocked by atipamezole and BRL-44408 (*p* < 0.01, group B1 or B3 vs. group D2) but not by ARC-239 (*p* > 0.05, group B2 vs. group D2; Figure 4A,B).

**Figure 5:**
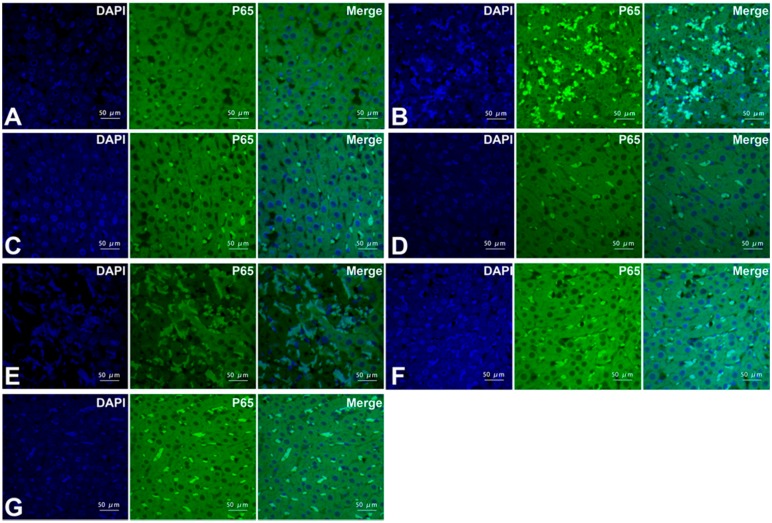
Expression of the phospho-NF-κB subunit p65 protein in liver tissue of the different groups as measured by immunofluorescence staining observed via laser scanning confocal microscopy (LSCM) (magnification, ×400, scale bars, 50 μm). (**A**) Group S; (**B**) group M; (**C**) group D1; (**D**) group D2; (**E**) group B1; (**F**) group B2; and (**G**) group B3. Positive p65 cells were stained green, and the sections were counterstained with 4,6-diamidino-2-phenylindole(DAPI) to visualize the nuclei.

**Figure 6:**
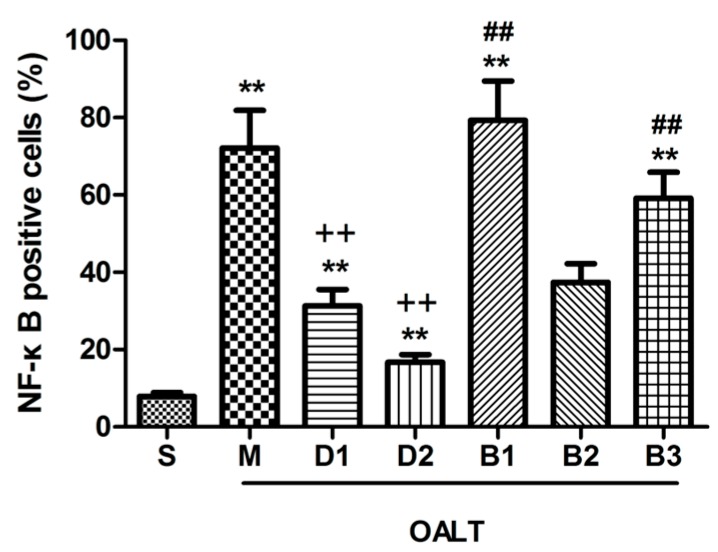
The ratio of phospho-NF-κB subunit p65-positive cells in the liver tissue of the different groups. Cells were counted via LSCM (magnification, ×400). The data are expressed as the means ± SD (*n* = 8). ** *p* < 0.01, vs. group S; ^++^ *p* < 0.01, vs. group M; ^##^ *p* < 0.01, vs. group D2.

Immunofluorescence-staining and Western blotting demonstrated that positive phospho-NF-κB subunit p65 cells were stained green and mainly located in the nuclei. Nuclear translocation levels of NF-κB is accelerated during OALT (*p* < 0.01, group M vs. group S); Dex significantly decreased NF-κB activation induced by OALT (*p* < 0.01, group D1 and D2 vs. group M), indicating that Dex probably inhibits the activation, nuclear transport or even transcription of the NF-κB genes/proteins. The effect of Dex on the NF-κB p65 was completely blocking by treatment with atipamezole and BRL-44408 (*p* < 0.01, group B1 and B3 vs. group D2) but not by ARC-239 treatment (*p* > 0.05, group B2 vs. group D2; Figure 4C,D, Figure 5 and Figure 6).

### 2.4. Dexmedetomidine Inhibits Neutrophil Infiltration and Pro-Inflammatory Cytokine Release during OALT

**Figure 7:**
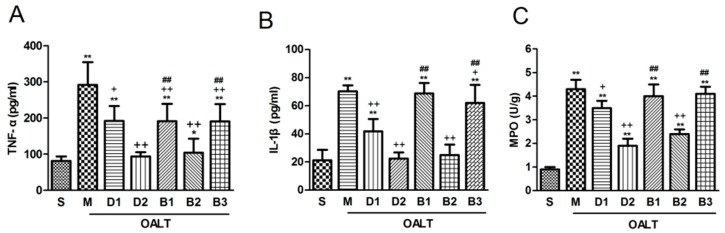
The concentrations of plasma TNF-α (**A**); IL-1β (**B**) and activity of hepatic myeloperoxidase (MPO) (**C**) in the different groups. The data are expressed as the means ± SD (*n* = 8). * *p* < 0.05, ** *p* < 0.01, vs. group S; ^+^ *p* < 0.05, ^++^ *p* < 0.01, vs. group M; ^##^ *p* < 0.01, vs. group D2.

As shown in Figure 7, Dex administered alone at 10 or 50 µg/kg significantly attenuated myeloperoxidase (MPO) activity in liver tissue and inhibited the release of serum pro-inflammatory mediators (TNF-α and IL-1β) in the serum (*p* < 0.05, group D1 or group D2 vs. group M). When combined with either the nonsubtype-specific α_2_ antagonist atipamezole or the α_2A_-specific antagonist BRL-44408, these anti-inflammatory effects of Dex were abolished, and the level of cytokines and MPO activity significantly increased (*p* < 0.01, group B1 or group B3 vs. group D2). However, following the co-administration with ARC-239, the Dex-induced anti-inflammatory effect did not change significantly (*p* > 0.05, group B2 vs. group D2).

## 3. Discussion

This study investigated whether Dex pretreatment produces a protective effect on the liver suffering IR injury relating to liver transplantation. In the rat model of OALT, we found that Dex, a novel highly-selective α_2_-AR agonist, attenuates OALT-induced hepatic injuries, probably by suppressing the TLR4/NF-κB pathway and decreasing inflammatory mediator levels. The protective effects of Dex were reversed by atipamezole (a nonspecific α_2_ receptor blocker) and/or BRL-44408 (a specific α_2A_ receptor blocker), but not by ARC-239 (a specific α_2B/C_ receptor blocker), indicating that these effects were mediated, at least in part, by activating the α_2A_-adrenoceptor subtype, and the mechanism is associated with the suppression of the TLR4/NF-κB pathway.

In the current study, we found that Dex significantly reduced pathological liver damage and improved liver function in rats undergoing OALT. Our data are consistent with evidence from previous studies. Dex decreases central venous congestion, and reduces inflammation of the portal tracts and dilation of the hepatic sinusoids in sepsis [17]. A study by Arslan et al. [18] discovered the ability of Dex to prevent malonaldehyde and sALT increases and erythrocyte deformability alterations in hepatic IR injury. Another investigation demonstrated that Dex protects rats against lipid peroxidation and histological damage in IR-induced injury to the liver and other remote organs (kidney and lung) [19].

In addition to its cytoprotective effects on the liver, our studies demonstrated that Dex suppressed the TLR4/NF-κB inflammatory signaling, as evidenced by the downregulation of the TLR4 and p65 proteins in the liver and the inhibited release of the pro-inflammatory cytokines (TNF-α and IL-1β) in the serum by Dex pre-treatment. TLR4 has been shown to be triggered through endogenous ligands, including damage-associated molecular patterns (DAMPs) and cytokines [6,7,8,9]. NF-κB is a key factor of nuclear transcription; that is, NF-κB will rapidly transfer into the nucleus upon activation, then amplify inflammatory cascades by promoting the production of pro-inflammatory cytokines (TNF-α, IL-1β, and IL-6). TLR4 plays a dominant role in the pathogenesis of liver IR injury via the activation of NF-κB signaling [7,8,9,10]. Evidence has revealed that the absence of *TLR4* genes in the liver reduces liver IR injury [6,7,8,9,10,11,12], and TLR4 blockade affects the function of hepatocytes and Kupffer cells, depresses the production of proinflammatory cytokines (TNF-α and IL-6) and ameliorates hepatocellular IR injury [6]. Some pharmacologic agents exert anti-inflammatory and hepatoprotective effects through the TLR4/NF-κB pathway, but these drugs have never been routinely implemented in clinics and are seldom applied peri-operatively in orthotopic liver transplantation [5,11,24]. Dex, a novel sedation and adjuvant in the operating room and critical care settings, was found to inhibit inflammatory reactions in the lung by suppressing the TLR4/NF-κB pathway in septic rats [2,15]. Gu et al. [16] found that Dex protects against IR injury to the kidney in mice and that the mechanism is due to the suppression of high-mobility group protein B1 (HMGB-1) release and the subsequent inhibition of TLR4 signaling. In this study, we found that Dex at 50 µg/kg significantly decreases both the expression of TLR4 and NF-κB p65 protein after OALT; although with NF-κB p65 downregulated, Dex at 10 µg/kg did not affect the increased expression of TLR4 during OALT, even increased its expression a little (Figure 4A,B). There are several potential factors attributing to these unanticipated findings: (1) it is difficult to ensure that the doses we selected can completely inhibit the expression of TLR4, and dose-dependent effect (i.e., 10, 20, 30, 40, and 50 µg/kg) of Dex should be investigated; (2) other organ-protective mechanisms may exist during low-dose Dex administrated [16,25]; (3) the sample size for measuring the TLR4 expression is not enough and the experimental repeatability is poor, which leads to the statistical errors.

The organ protective mechanism of Dex is still not clear but may be largely attributable to anti-oxidant, anti-inflammatory (as shown above), and other cytoprotective properties via the activation of α_2_-ARs. Most α_2_-AR agonists mediate their physiological and pharmacological actions mainly via the activation of α_2_-ARs and the modulation of catecholamine (norepinephrine and epinephrine) release. α_2_-ARs are widely known as Gi- and Go-protein-coupled receptors that decrease intracellular cAMP, inhibit voltage-gated Ca^2+^ channels, while open K^+^ channels, which leads to the hyperpolarization of neurons and has inhibitory effects on neurotransmitter release [26]. In the central nervous system (CNS), α_2_-ARs are predominantly located pre-synaptically. These receptors modulate the release of catecholamines through a negative feedback mechanism. In the periphery, α_2_-ARs are widely distributed in vital organs and blood vessels [20]. Evidence has indicated that catecholamines lead to an increase of oxygen free radical and an overload of Ca^2+^, eventually resulting in cell injury [27]. In addition, norepinephrine was found to modulate the responsiveness of macrophages to proinflammatory mediators through the activation of the α_2_-AR [28], and induce hepatocellular dysfunction by the activation of the α_2_-AR in early sepsis [29]. In this study, the protective effects of Dex were reversed by atipamezole (a complete α_2_-AR antagonist); therefore, we presumed that Dex prevents liver IR injury by inhibiting catecholamine release in an α_2_-AR-dependent manner.

Three subtypes of α_2_-ARs (α_2A_, α_2B_, and α_2C_) were identified by pharmaceutical interventions, and were cloned from several species including rats and humans [20,21]. Experiments with transgenic engineered rats suggest that most of the effects of α_2_-AR agonists are mediated by the α_2A_-adrenoceptor subtype predominantly located pre-synaptically in the CNS [21]. In contrast, the role of α_2B_- and α_2C_-adrenoceptors seems to be much more restricted. The α_2B_-adrenoceptor subtype has been shown to be essential for placental vascular and lung development and temporary vasoconstriction. The α_2C_-adrenoceptor subtype was found to mediate different behavioral functions and control the release of epinephrine from the adrenal gland [26].

In the current study, using pre-intervention with three α_2_-AR subtype antagonists, we found that the hepatic protective effect of Dex probably occurs via the α_2A_ subtype rather than the α_2B_ or α_2C_ subtypes, and this result has been confirmed by previous investigations using either pharmacological intervention or transgenic methods. Recent work by Ma et al. [22] who utilized α_2_-AR subtype antagonists similar to those mentioned in our experiment, suggests that Dex affords its neuroprotective effect by activating the α_2A_-adrenoceptor subtype in a rat model of brain hypoxia-ischemia. By the transgenic knockout of the α_2A_-adrenoceptor (α_2A_-KO) or α_2C_-adrenoceptor (α_2C_-KO) subtypes, Andrea et al. [23] consistently found that Dex exerts potent neuroprotection in either wild-type mice or α_2C_-KO mice, while not in α_2A_-KO mice, which suggested that this neuroprotective effect is mediated by the α_2A_-adrenoceptor subtype.

The role of the α_2A_ subtype in the processes of systemic inflammatory cascades and sepsis has been well studied. In a series of animal studies relating to early sepsis, the α_2A_-adrenoceptor subtype was proven to be located on hepatic macrophages (i.e., Kupffer cells) in liver tissue [28,30], and α_2A_-adrenoceptor gene expression was significantly increased 2 h after cecal ligation and puncture (CLP), while no significant changes were observed in the α_2B_ or α_2C_ subtypes [30]. Another investigation found that a blockade of the α_2A_-adrenoceptor with BRL-44408 suppresses early pro-inflammatory mediators (TNF-α, IL-6, etc.) and, thus, improves liver dysfunction during sepsis [31]. Furthermore, α_2A_-adrenoceptor blockade also improves sepsis-induced acute lung injury (ALI) accompanied by depressed levels of HMGB-1 in rats [32]. These findings provide evidence that the activation of the α_2A_ subtype may promote a sepsis-like inflammatory cascade during liver IR injury. However, in this study, we found that blocking the α_2A_ subtype with BRL-44408 reversed the anti-inflammatory effect of Dex; the inflammatory cascade was thus amplified. We speculate that Dex may activate the pre-synaptic α_2A_-adrenoceptor subtype expressed on Kupffer cells or other sites, and then inhibit norepinephrine release via a cAMP-dependent manner, thereby downregulating the inflammatory response and attenuating liver damage during OALT. Nevertheless, the relationship between the activation of α_2A_-AR subtype and the suppression of TLR4/NF-κB pathway is not clear. They may act independently, or synergistically. Both α_2A_-adrenoceptor and TLR4 are transmembrane receptors, and the specific interaction between them requires further research.

There are several possible limitations to this study. First, more negative control groups are required to determine whether the doses of the agents that we employed actually possessed hepato-toxic or hepato-sparing effects. Second, reagent ARC-239 displays a high affinity for the α_2B_ and α_2C_ subtypes with no selectivity [33]; therefore, more specific antagonists, as well as transgenic animals targeting the genes of the three α_2_-AR subtypes, are necessary. Third, the distribution and localization of the α_2_-AR subtypes in the liver remain to be verified. Finally, Dex also exhibits some affinity for imidazoline binding sites [34], and the imidazoline receptor may be involved in the protective effect of Dex [25].

## 4. Materials and Methods

### 4.1. Animals and Surgical Procedure

Fifty-six adult, pathogen-free, male Sprague-Dawley rats, weighing 200–250 g, were purchased from the Medical Experimental Animal Center of Guangdong Province, China. All experimental protocols were performed in accordance with the institutional criteria for the care and use of laboratory animals in research. All animals were provided with standard chow and sterile acidified water and were housed in temperature- and humidity-controlled cages in accordance with institutional animal care policies. Food was removed 8 h before the animals were used, but they continued to have free access to water.

The orthotopic autologous liver transplantation (OALT) model was established as described [13,14,35]. Briefly, under anesthesia, the ligaments, vessels, and bile ducts around the liver were carefully dissociated, and the entire liver was well exposed. Four vessels, including the super-hepatic vena cava (SHVC), inferior hepatic vena cava (IHVC), hepatic artery (HA), and portal vein (PV) were clearly anatomized. Before the occlusion of these vessels, approximately 50 units of heparin (diluted with saline) was injected via the tail vein, and a cannula was then inserted into the PV. With these preparations, the HA and PV were clamped with atraumatic hemostatic clips, followed by the occlusion of the SHVC and IHVC blood flow. The liver was then irrigated with 250 U of cold (2–4 °C) heparin at a rate of 2.0 mL/min through the PV catheter, and a 1.0-mm hole was made in the wall of the IHVC as an outflow tract. The entire ischemia time allowed was 20 min. Finally, the openings in the PV and IHVC were repaired using 8-0 sutures, and the PV, SHVC, IHVC, and HA were unclamped.

All the rats were sacrificed at 8 h of reperfusion, and liver tissues and blood samples were collected. Serum was separated and stored at −80 °C until analysis. Liver tissues were fixed in 10% formalin for histological analysis or frozen immediately in liquid nitrogen for biochemical assays.

### 4.2. Grouping and Drug Treatment

Animals were randomly allocated into seven groups (*n =* 8 each) as follows.

(1) Sham group (group S) rats subjected to abdomen dissection and isolation of the hepatic peripheral vessels without occlusion;
(2) Model group (group M) rats underwent the OALT procedure as described above, and no drug was utilized;
(3) Low-dose Dex group (group D1) and high-dose Dex group (group D2) rats received 10 or 50 μg/kg Dex (Hengrui Pharmaceutical Co., Ltd., Nanjing, China), respectively, via intraperitoneal injection 30 min before the operation; and
(4) Atipamezole + high-dose Dex group (group B1), ARC-239 + high-dose Dex group (group B2), and BRL-44408 + high-dose Dex group (group B3) rats received 500 μg/kg atipamezole (a nonspecific α_2_ receptor blocker, Sigma-Aldrich, St. Louis., MO, USA), 50 μg/kg ARC239 (a specific α_2B/C_ receptor blocker, Santa Cruz Biotechnology, Santa Cruz, CA, USA), or 1.5 mg/kg BRL-44408 (a specific α_2A_ receptor blocker, Sigma-Aldrich), respectively, via intraperitoneal injection 10 min before receiving 50 μg/kg Dex 30 min prior to the OALT.

In the current study, all the drugs were dissolved in normal saline. Based on previous studies, Dex was administered 30 min before or immediately after the liver IR injury [16,19]. The dose selected for each antagonist was on the basis of the antagonist’s affinity and the dose-effect relationship with Dex.

### 4.3. Histological Evaluation

Tissue specimens were fixed in 10% formalin for 48 h and then embedded in paraffin and cut into 5-mm sections. The slides were stained with hematoxylin/eosin and then analyzed blindly using Suzuki’s criteria of liver damage [36]. In this classification, sinusoidal congestion, hepatocyte necrosis and ballooning are graded from 0 to 4. No necrosis, congestion or ballooning is given a score of 0, whereas severe congestion or ballooning, degeneration and >60% lobular necrosis are given a value of 4.

### 4.4. Serum Alanine Aminotransferase Detection

Serum alanine aminotransferase (sALT), an indicator of hepatocellular injury, was measured using an Olympus AU640 autoanalyzer (Diamond Diagnostics, Watford, UK). The sALT results were expressed as units per liter.

### 4.5. Western Blot Analysis

To investigate whether Dex exerts its effects through TLR4/NF-κB/p65 pathways, the expression of TLR4 and NF-κB/p65 proteins were detected via Western blot. The liver tissues were homogenized on ice, diluted with 10 volumes of natural saline, and then centrifuged at 2500 rpm for 10 min. The supernatants were transferred into fresh tubes for biochemical analysis. Nuclear and cytoplasmic proteins were extracted by using nuclear and cytoplasmic extraction reagents according to the manufacture’s procedure (Nanjing Jiancheng Biologic Product, Nanjing, China). Samples containing 30 μg of extracted protein were loaded onto a 10% SDS-PAGE premade gel (Invitrogen, Paisley, UK) for electrophoresis and then transferred to a polyvinylidene fluoride membrane pre-treated with 100% methanol. Subsequently, the membrane was blocked with PBS containing 5% non-fat milk for 1 h at 37 °C and then incubated with rabbit anti-TLR4 polyclonal antibody (1:2000, Santa Cruz Biotechnology), the rabbit polyclonal anti-NF-κB p65 antibody (1:500, Santa Cruz Biotechnology), the rabbit polyclonal anti-Histone H3.1 polyclonal antibody (1:500, Santa Cruz Biotechnology), and anti-β-actin antibody (1:1000, Santa Cruz Biotechnology) for 2 h at 37 °C followed by incubation with rat monoclonal secondary antibody directed against the primary antibody for 1 h at 37 °C. The results of TLR4 and NF-κB p65 were normalized with respect to β-actin and Histone H3.1 band density.

### 4.6. Immunofluorescence Assay

Paraffin-embedded sections of liver tissue were dewaxed and rehydrated. After 3 h of antigen retrieval in 10 mM of sodium citrate (pH 6.0), the sections were incubated with blocking buffer (5% BSA in PBS) for one hour at room temperature; Then, the sections were incubated with a primary rabbit anti-phospho-NF-κB p65 antibody (Cell Signaling Technology, Danvers, MA, USA) at a dilution of 1:100 overnight at 4 °C. The sections were washed with PBS and then incubated with a fluorescein isothiocyanate (FITC)-conjugated goat anti-rabbit secondary antibody at a dilution of 1:100 (Life Technologies, Carlsbad, CA, USA) for 2 h at room temperature in the dark. The sections were counterstained with DAPI to visualize nuclei and then examined using a laser scanning confocal microscopy (LSCM) (Zeiss LSM 510 META, Jena, Germany).

### 4.7. Enzyme-Linked Immunosorbent Assay (ELISA)

Serum concentrations of TNF-α and IL-1β were measured using commercially available ELISA kits (USCN Life Science Inc., Wuhan, China) according to the protocols provided by the manufacturer. Pro-inflammatory cytokine concentrations were expressed as picograms per milliliter (pg/mL).

### 4.8. Myeloperoxidase (MPO) Activity

The presence of MPO was used as an index of neutrophil accumulation [37]. All of the reagents were purchased as a kit from Nanjing Jiancheng Biology Engineering Institute. Briefly, liver tissue was placed in 0.5% hexadecyltrimethyl-ammonium bromide and 50 mM potassium phosphate buffer solution (pH = 5.0). Each sample was homogenized and centrifuged, and the supernatant was allowed to react with a solution of 1.6 mM tetramethylbenzidine and 0.1 mM H_2_O_2_. The change in absorbance was measured with a spectrophotometer at 460 nm. MPO activity was defined as the quantity of enzyme degrading 1 mmol of peroxide per minute at 37 °C and was expressed in units per gram of wet tissue.

### 4.9. Statistical Analysis

All experimental results were expressed as the means ± SD. Analyses were performed with the SPSS 13.0 Statistical Software (SPSS Inc, Chicago, IL, USA) for Windows. Statistical comparisons were performed using the one-way analysis of variance (ANOVA) method followed by a *post hoc* Bonferroni’s *t*-test where appropriate. A two-tailed *p* value less than 0.05 was considered statistically significant.

## 5. Conclusions

In summary, the results of this study suggest that Dex pretreatment attenuates OALT-induced liver injury partly via the α_2A_-adrenoceptor subtype, and the mechanism is due to the suppression of the TLR4/NF-κB pathway. However, the interactions between α_2A_-AR subtype and TLR4/NF-κB pathway are unclear. Further studies to explore this subject will be required.

## Abbreviations

α_2_-AR	α_2_-adrenoceptor
α_2A_-KO	α_2A_-adrenoceptor knockout
α_2B_-KO	α_2B_-adrenoceptor knockout
α_2C_-KO	α_2C_-adrenoceptor knockout
CNS	central nervous system
DAMPs	damage-associated molecular patterns
DAPI	4,6-diamidino-2-phenylindole
Dex	dexmedetomidine
HA	hepatic artery
HMGB1	high-mobility group protein B1
IHVC	inferior hepatic vena cava
LSCM	laser scanning confocal microscopy
MPO	myeloperoxidase
NF-κB	nuclear factor kappa B
OALT	orthotopic autologous liver transplantation
PV	portal vein
sALT	serum alanine aminotransferase
SHVC	super-hepatic vena cava
TLR	toll-like receptor
TLR4	toll-like receptor 4
